# Branched‐chain fatty acids in the vernix caseosa and meconium of infants born at different gestational ages

**DOI:** 10.1002/fsn3.2306

**Published:** 2021-05-06

**Authors:** Weidi Li, Liang Jie, Renqiang Yu, Qingzhe Jin, Shanyu Jiang, Qitao Yin, Wei Wei, Xingguo Wang

**Affiliations:** ^1^ Collaborative Innovation Center of Food Safety and Quality Control in Jiangsu Province School of Food Science and Technology Jiangnan University Wuxi China; ^2^ The Affiliated Wuxi Maternity and Child Health Care Hospital of Nanjing Medical University Wuxi China; ^3^ State Key Laboratory of Dairy Biotechnology Shanghai Engineering Research Center of Dairy Biotechnology Synergetic Innovation Center for Food Safety and Nutrition Dairy Research Institute Bright Dairy & Food Co., Ltd. Shanghai China

**Keywords:** branched‐chain fatty acids, fatty acid composition, gestational age, meconium, vernix caseosa

## Abstract

The functional lipid components found in breast milk, vernix caseosa, and meconium are Branched‐chain Fatty Acids (BCFA). The goal of this study was to establish the existence of BCFA in vernix and meconium in infants born at different gestational ages. TLC plates and GC‐MS were examined for the lipids in vernix caseosa and meconium. The results indicated that there were nine BCFA in vernix caseosa, including *iso*‐12:0, *anteiso*‐13:0, *iso*‐14:0, *iso*‐15:0, *anteiso*‐15:0, *iso*‐16:0, *anteiso*‐17:0, *iso*‐18:0, and *iso*‐20:0. Five BCFA (*iso*‐12:0, *anteiso*‐13:0, *iso*‐14:0, *iso*‐15:0, and *anteiso*‐15:0) were not contained in the meconium, suggesting that some of the BCFA may be digested and consumed by infants. In the vernix caseosa, the content of BCFA in triacylglycerol (TAG) and free fatty acid (FFA) was 15.59% and 11.82%, respectively. The vernix caseosa's wax ester fraction contained the highest content of BCFA, reaching up to 16.81%. The carbon chain length of fatty acids (FA) ranged from 12 to 24 in the vernix caseosa and 14 to 22 in meconium samples. The gestational age was likely to affect BCFA concentrations, with the vernix caseosa and meconium BCFA content being significantly higher in full‐term infants than in preterm infants (*p* < .001). Further research is required into the relationship between BCFA and gut microbiotas.

## Graphical abstract

The content of BCFA in the vernix and meconium was significantly higher in full‐term infants than in preterm infants. BCFA may be digested and absorbed in the infants’ gastrointestinal tract.

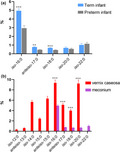

## 1 INTRODUCTION

During the last three months of pregnancy, the sebaceous glands in the skin of infants synthesize the vernix caseosa. Infants beginning at 28 weeks of gestation are densely coated. The vernix is suspended in amniotic fluid in the later phases of pregnancy and is swallowed to receive nutrients by the fetus. In addition to approximately 80.5% of water and some epithelial cells, vernix caseosa is composed of about 9.1% proteins and 10.3% lipids (Haubrich, 2003; Rissmann et al., 2006; Yan et al., 2018). The content of branched‐chain fatty acids (BCFA) is especially rich among the fatty acids (FA), accounting for 10%–20% of the dry weight of vernix caseosa (Ran‐Ressler et al., 2008a).

Branched‐chain Fatty Acids are predominantly saturated fatty acids (SFA), which include monomethyl, dimethyl, or polymethyl BCFA with one or more methyl branching point on the carbon chain, and the conspicuous branching is near the trailing end of the carbon chain (Dingess, Valentine, Ollberding, Davidson, Woo, Summer, Peng, Guerrero, Ruiz‐Palacios, Ran‐Ressler, et al., 2017; Ran‐Ressler et al., 2014).

In the membranes of a large variety of Gram‐positive bacteria such as Lactobacilli, Mycobacterium, and Bifidobacteria, BCFA are the popular FA (Ran‐Ressler et al., 2014; Veerkamp, 1971). In fact, bacterial lipids are typically composed of polar lipids, while food lipids are dramatically made up of triacylglycerols (TAG) (Hauff & Vetter, 2010; Yan et al., 2017). Dairy products are the most common kind of food rich in BCFA, and their content has a certain relationship with milk source.

While BCFA are normal in nature, they are not abundant in the adult diet (Dingess, Valentine, Ollberding, Davidson, Woo, Summer, Peng, Guerrero, Ruiz‐Palacios, & Ran‐Ressler, 2017); however, infants have been found to swallow 200–500 ml/d of amniotic fluid near term (Ran‐Ressler et al., 2008b), containing approximately 6 mg BCFA. Indeed, BCFA have also been detected in the meconium, which is the first fecal matter from infants, starting at around the 12th week of gestation (Ostrea et al., 2006; Ran‐Ressler et al., 2008b). The proportion of BCFA in total FA in the meconium can be as high as 15% (0.75 mg/100 mg) (Terasaka et al., 1986).

After investigating the BCFA present in breast milk at different lactation periods in Chinese patients (Jie et al., 2018), we studied the FA from the vernix caseosa of full‐term and preterm infants, with an emphasis on BCFA and their comparison with FA from the meconium. Yan et al. (2017) studied the distribution of BCFA on TAG and found that BCFA are enriched in the *sn*‐2 position of human milk, in contrast, BCFA randomly distributed among the *sn*‐2 and *sn*‐1/3 positions of cow and goat milk (Qi et al., 2018). BCFA are trace but essential elements, which have special nutritional, healthcare functions, and pharmacological activities due to their special branched‐chain structure. However, predecessors reported more on BCFA in dairy products; there are scant data focused on the BCFA in the vernix caseosa. Our findings may enhance the understanding of lipids in the infant gastrointestinal tract and intestines before birth and may encourage additional competitive studies on the association of these lipids in early life with the gut microbiome.

## 2 METHODS AND MATERIALS

### 2.1 Chemicals

Branched‐chain Fatty Acids standards 12‐methyltridecanoic acid (*iso*‐14:0), 12‐methyltetradecanoic acid (*anteiso*‐15:0), 14‐methylpentadecanoic acid (*iso*‐16:0), and 14‐methylhexadecanoic acid (anteiso‐17:0) were purchased from Larodan Fine Chemicals. Hexane of high‐performance liquid chromatography (HPLC) grade was purchased from J&K Scientific Ltd. Silicic acid thin‐layer chromatography (TLC) plates (20 cm × 10 cm, or 20 cm × 20 cm, layer thickness: 1.0 mm) were purchased from Sigma‐Aldrich. Solid Phase Extraction (SPE) Cartridges (200 g, 3 ml) were purchased from Zhenjiang Jiedao Instrument Technology Co., Ltd. Chloroform, methanol, and diethyl ether of analytical purity, acetyl chloride, sodium sulfate, anhydrous, sodium carbonate, and 2,6‐Di‐terc‐butyl‐4‐methylphenol (BHT) were purchased from Sinopharm Chemical Reagent Co. Ltd. Ethanol was purchased from Jiangsu Bomeida Life Science Co. Ltd.

### 2.2 Samples collection

The vernix caseosa was wiped from the newborn's skin, and the first meconium was collected according to Rissmann et al. (2006). All experimental samples (vernix caseosa and meconium) were provided by the Wuxi Maternal and Child Health Hospital (Wuxi, China) and stored at −80°C in a freezer until further analyses. All experiments were approved by the Ethics Committees of the Medical Research Board of Jiangnan University and Wuxi Maternity and Child Health Care Hospital (WXM201695).

Seventeen vernix and meconium samples were collected, including preterm infants (29–36 weeks, *n* = 11) and full‐term infants (≥37 weeks, *n* = 6). The maternal age of the mother of preterm infants and the mother of term infants was 30.45 ± 2.98 years and 29.17 ± 4.22 years. With regard to the type of delivery, 36.36 percent of mothers of infants and 66.67 percent of mothers of infants chose eutocia. The gestational age of preterm infants and term infants was 237.55 ± 14.44 days and 272.50 ± 7.92 days, respectively. The parity of preterm infants' mothers and term infants' mothers was 0.18 ± 0.40 and 0.50 ± 0.55, respectively. The proportion of male preterm infants and term infants was 81.82% and 66.67%. The birth weight of preterm infants and term infants was 2.15 ± 0.40 kg and 2.57 ± 0.23 kg, respectively.

### 2.3 Lipid extraction

Lipids were extracted as described by Míková (Kaneda, 1977; Míková et al., 2014). Gauze and diapers containing the vernix caseosa and meconium were dissolved in 50 ml of chloroform/methanol (2:1, v/v) with 0.05% of BHT. The suspension was ultrasound for 40 min and filtered by SPE column (60–120 mm, 0.2 g). Trace amount of water was removed by adding anhydrous Na_2_SO_4_ (approximately 5 g), and the suspension was filtered by SPE column. The solvent was removed by nitrogen. The lipids were stored at −20°C until further analysis.

### 2.4 Lipid class analysis

The lipids (100 mg) were separated using hexane/diethyl ether (93:7, v/v) as a mobile phase on a 20 cm × 10 cm silicic acid TLC plates. Each TLC plate has been developed twice to improve lipid separation. Different lipid classes were distributed visualizing in iodine tank (Míková et al., 2014). The retention factor (R*f*) values of different lipid classes were calculated according to standards and reference (Rissmann et al., 2006), which were squalene (R*f* 0.89–0.94), wax esters and cholesteryl esters in one zone (R*f* 0.66–0.74), diol diesters (R*f* 0.46–0.52), TAG (R*f* 0.19–0.27), free fatty acids (FFA, R*f* 0.10–0.13), cholesterol (R*f* 0.06–0.08), and polar lipids (R*f* 0.00–0.01). Each lipid band was scraped off from the TLC plate and added into the diethyl ester. After centrifugation, take the supernatant liquid to remove the solvents by nitrogen evaporated. By the weighing of each band, the lipid content was determined (Kärkkäinen et al., 1965). The lipids were stored −80°C until further analysis.

### 2.5 Composition analysis of BCFA in *sn*‐2 monoacylglycerols (*sn*‐2 MAG)

The analysis of *sn*‐2 MAG FA was based on the improved method described by Luddy et al. (1964). In brief, 7 ml of 1 M Tris–HCl buffer (PH = 8), 1.75 ml of 0.05% bile salts, 0.7 ml of 2.2% CaCl_2_ solution, and 30 mg pancreatic lipase were applied to the extracted lipids. The mixture was incubated for 3 min at 37°C and shaken for 30 s. These steps were repeated three times, then 2 ml of ether was added, and the mixture was centrifuged (1800 *g*, 5 min). The ether layer was desiccated with anhydrous Na_2_SO_4,_ and evaporated to 200 μl under nitrogen. The ether layer hydrolysate was isolated on the TLC plate, to be unfolded using hexane, ether, and ethyl (50:50:1, v/v/v). The band corresponding to *sn*‐2 MAG was scraped off, then methylated, and analyzed by GC‐MS.

### 2.6 Methylation of FA and GC analysis

The collected lipid samples were methylated, according to Míková et al. (2014). Samples were dissolved into chloroform/methanol (2:3, v/v) solution. Fatty acid methyl esters (FAMEs) were obtained via methylation with acetyl chloride (1 ml) at 70°C for 60 min. Trace amounts of water were removed from FAME extracts with anhydrous Na_2_SO_4_, and FAMEs were then dissolved in *n*‐hexane.

Samples were analyzed using a DB‐5 column‐equipped SCIONSQ‐456 GC‐MS (Bruker Instruments Company) (30 m × 0.25 mm × 0.10 m, Agilent Technologies) as described previously (Jie et al., 2018). The temperature schedule has been set for 5 min at 150°C; rise to 210°C with a heating rate of 3°C/min and hold for 2 min at this temperature; rise to 260°C with a heating rate of 10°C/min and hold for 5 min at this temperature. The temperature of the injector was set at 280°C with a division ratio of 1:100. It was injected with 1 μl of sample solution. As the carrier gas, the flow velocity of high purity helium (99.999%) was 1.2 ml/min. The temperature of Mass spectrometry was 230°C with the electron energy of 70 eV. The transmission line temperature was 250°C. Mass spectrometry used full‐scan mode with a scan range from 45 to 650 m/z, and the solvent delay time was 5 min. In order to describe FA, we used generic FA standards and spectra library.

### 2.7 Statistical analysis

Data treatment was performed using IBM SPSS Statistics 19 (Armonk, New York, USA). Statistical significance (*p* < .05) was analyzed by the Student's *t* test or analysis of variance (ANOVA). Graphing was performed using GraphPad Prism 8.

## 3 RESULTS

### 3.1 Lipids in vernix caseosa and meconium

Both vernix caseosa and meconium, which accounted for 11.2% and 7.3%, were extracted from the lipid fractions. As shown in Table 1, the most abundant lipids in the meconium were squalene (20%), wax esters, cholesterol esters (51%), TAG (15%), and fatty alcohol (14%). The vernix caseosa contained squalene (17%), wax esters, and cholesterol esters (25%), TAG (20%), as well as small amounts of polar lipids, fatty alcohol (12%), and FFA (15%).

**TABLE 1:** Profile of lipids in the vernix caseosa and meconium of term infants

Lipid class	Infant vernix caseosa (%)	Infant meconium (%)
Wax esters and cholesterol esters	25	51
Triacylglycerols	20	15
Squalene	17	20
Diol diesters	11	nd
Free fatty acid	15	nd
Fatty alcohol	12	14

Note

The standard deviations of the three determinations were estimated to be ≤5%.

### 3.2 FA composition in vernix caseosa and meconium

A total of 23 FA were identified in the vernix caseosa of both preterm and term infant samples using GC‐MS, and the FA carbon chain length ranged from 12 to 24. Table 2 shows that the FA composition in the vernix caseosa of preterm and term infants was very different. Lipids in the vernix caseosa of preterm infants are rich in the sum of SFA and the sum of polyunsaturated fatty acids (PUFA), for example, 16:0 and 18:2 n‐6. At the same time, the FA were higher in the term infants' lipids. The concentration of all BCFA in preterm infants' vernix was significantly lower (*p* < .001) than in term infants', 25.29 ± 0.51% and 43.02 ± 1.87%, respectively. The findings were close to the content of BCFA stated by Ran‐Ressler et al. (2008b), and the composition of lanolin FA (Wang et al., 2018).

**TABLE 2:** Fatty acid composition (wt%) in the vernix caseosa of preterm and term infants

FAs	Preterm infants	Term infants
12:0	0.26 ± 0.08	0.44 ± 0.09
13:0	0.09 ± 0.03	0.22 ± 0.07^*^
14:0	3.10 ± 0.19	4.21 ± 0.14^**^
15:0	2.81 ± 0.27	5.74 ± 0.47^***^
16:0	26.16 ± 1.14	5.91 ± 1.69^***^
17:0	4.45 ± 0.41	4.77 ± 1.04
18:0	16.28 ± 0.54	7.35 ± 1.23^***^
22:0	4.63 ± 0.13	2.68 ± 0.22^***^
24:0	3.44 ± 0.17	4.54 ± 1.33
14:1 *n*‐9	0.13 ± 0.03	0.18 ± 0.02
16:1 *n*‐7	0.43 ± 0.08	2.51 ± 0.34^***^
16:1 *n*‐9	2.47 ± 0.20	7.22 ± 1.10^**^
18:1 *n*‐9	nd	6.74 ± 3.67
18:2 *n*‐6	10.50 ± 0.42	4.51 ± 0.02^***^
*iso*‐12:0	0.20 ± 0.02	0.33 ± 0.08^*^
*iso*‐14:0	3.54 ± 0.31	5.67 ± 0.33^**^
*iso*‐15:0	1.74 ± 0.49	2.44 ± 0.17
*iso*‐16:0	7.59 ± 0.00	9.35 ± 0.55^**^
*iso*‐18:0	1.43 ± 0.63	4.00 ± 0.25^**^
*iso*‐20:0	4.65 ± 0.40	9.21 ± 0.69^***^
*anteiso*‐13:0	0.31 ± 0.06	0.60 ± 0.10^*^
*anteiso*‐15:0	3.80 ± 0.41	6.32 ± 0.44^**^
*anteiso*‐17:0	2.06 ± 0.27	5.12 ± 0.29^***^
∑SFA	61.20 ± 1.22	35.83 ± 3.23^***^
∑MUFA	3.03 ± 0.31	16.64 ± 5.09^**^
∑PUFA	10.50 ± 0.42	4.51 ± 0.02^***^
∑*iso*‐BCFA	19.13 ± 0.22	30.99 ± 1.25^***^
∑*anteiso*‐BCFA	6.16 ± 0.73	12.04 ± 0.63^***^
∑BCFA	25.29 ± 0.51	43.02 ± 1.87^***^

Note

Data are represented as mean ± *SD* (*n* = 3); nd, not detected.

*Significant differences from specific fatty acids in preterm infants: ^*^ *p* < .05; ^**^ *p* < .01; ^***^ *p* < .001.

The FA composition of vernix caseosa wax ester, TAG, the *sn*‑2 position of TAG, and FFA is shown in Table 3, in which BCFA accounted for 16.81%, 15.59%, 14.42%, and 11.82%, respectively, of the total FA in term infants. This indicated that BCFA were distributed uniformly in different vernix caseosa lipid forms. The wax ester fraction contained a higher content of unsaturated fatty acids (UFA), specifically 16:1 (41.46%). like human breast milk and freshwater fish oil, 16:0 was the predominant FA (33.38%) at the *sn*‐2 position of the vernix caseosa TAG.

**TABLE 3:** Comparison of fatty acids (wt%) in the meconium between preterm and term infants

	Preterm infants	Term infants
14:0	2.38 ± 0.09	2.28 ± 0.04
16:0	31.28 ± 0.66	32.44 ± 1.98
17:0	0.45 ± 0.05	0.84 ± 0.10^**^
18:0	15.08 ± 0.75	11.17 ± 0.28^**^
20:0	0.14 ± 0.02	0.71 ± 0.19^**^
22:0	0.28 ± 0.04	0.30 ± 0.03
16:1 n‐7	1.45 ± 0.04	0.94 ± 0.11^**^
18:1 n‐6	0.96 ± 0.03	2.73 ± 0.15^***^
18:1 n‐9	26.71 ± 0.71	20.20 ± 0.97^***^
18:2 n‐6	12.84 ± 0.4	16.92 ± 0.32^***^
22:5 n‐6	1.01 ± 0.07	1.21 ± 0.02^**^
22:6 n‐3	1.77 ± 0.11	2.17 ± 0.12^*^
iso‐16:0	3.00 ± 0.22	4.96 ± 0.15^***^
*iso*‐18:0	0.43 ± 0.03	0.68 ± 0.03^***^
*iso*‐20:0	0.58 ± 0.07	0.68 ± 0.08
*iso*‐22:0	1.18 ± 0.12	1.02 ± 0.13
*anteiso*‐17:0	0.47 ± 0.03	0.77 ± 0.08^**^
∑SFA	49.61 ± 1.28	47.73 ± 1.43
∑MUFA	31.9 ± 0.66	27.25 ± 0.91^**^
∑PUFA	12.84 ± 0.4	16.92 ± 0.32^***^
∑*iso*‐BCFA	5.19 ± 0.25	7.33 ± 0.14^***^
∑*anteiso*‐BCFA	0.47 ± 0.03	0.77 ± 0.08^**^
∑BCFA	5.66 ± 0.22	8.11 ± 0.22^***^

Note

Data are represented as the mean ± *SD* (*n* = 3).

*Significant differences from specific fatty acids in preterm infants: ^*^ *p* < .05; ^**^ *p* < .01; ^***^ *p* < .001.

### 3.3 Distribution of BCFA in meconium

**FIGURE 1:**
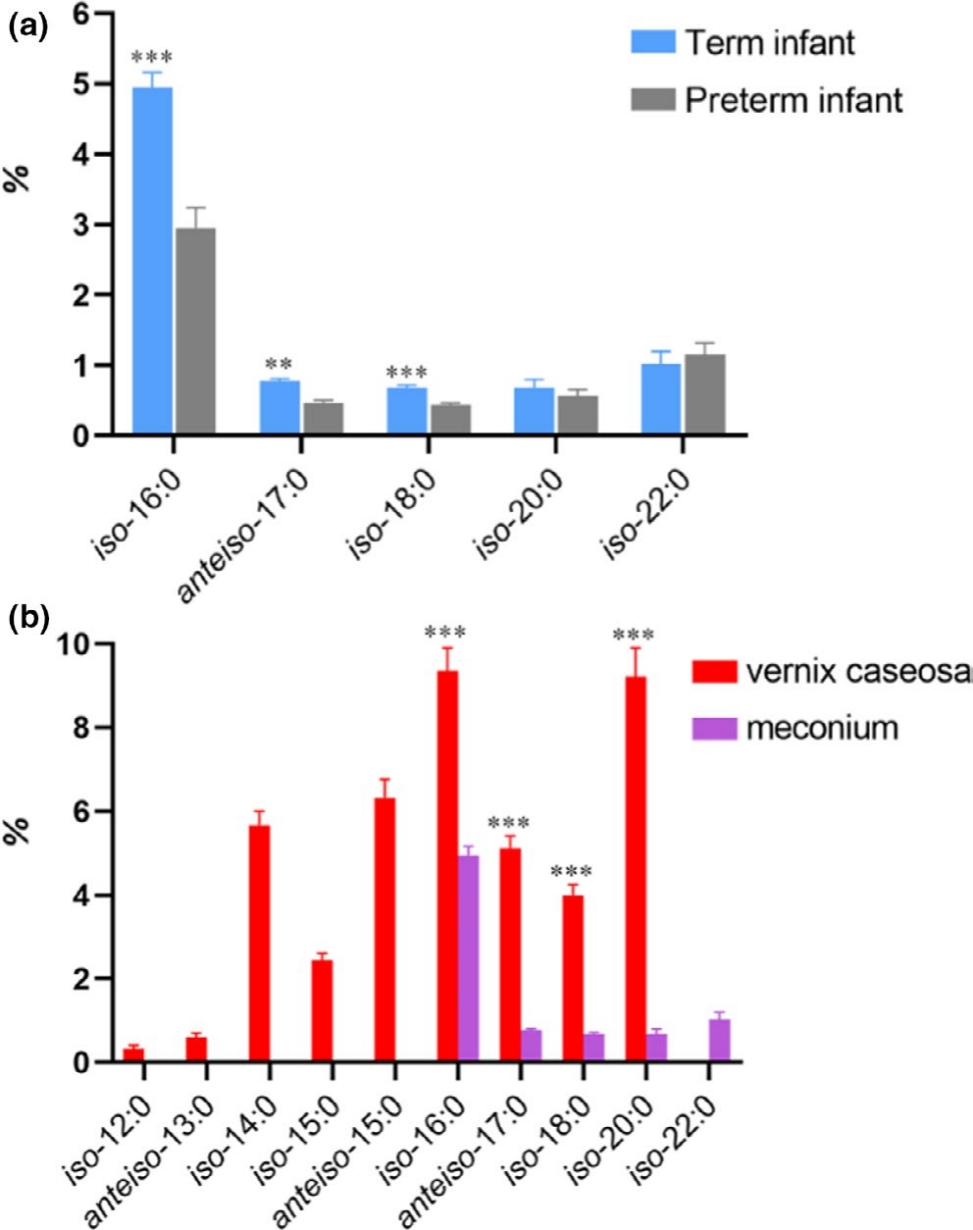
Composition of BCFA (wt%) in the meconium of term and preterm infants (a), and in the vernix caseosa and meconium of term infants (b)

As shown in Table S1, 17 types of FA were measured in meconium samples, with a chain length variation between 14 and 22 carbons. The composition of SFAs in preterm infants was almost the same as term infants'; 16:0 (~30%), 18:1 n‐9 (~20%), and 18:0 (~10%) were most abundant in both preterm and term infants. Only five types of BCFA, including one *anteiso*‐BCFA, were detected in the meconium, which accounted for 5.66 ± 0.22% and 8.11 ± 0.22% in preterm and term infants. Figure 1a shows the comparison of BCFA in the meconium of preterm and term infants. The concentration of BCFA with long carbon chains (*iso*‐16:0, *iso*‐18:0, and *anteiso*‐17:0) in the meconium of preterm infants was significantly lower than in term infants (*p <* .*01*), which was in agreement with a previous report (Ran‐Ressler et al., 2008b).

The results indicated that there were significant differences between the FA composition in the vernix caseosa and meconium. In contrast to the vernix caseosa, the types and contents of most BCFA in the meconium of term infants were less than those in the vernix caseosa. The concentration of *iso*‐16:0, *iso*‐18:0, and *anteiso*‐17:0, *iso‐*20:0 in the meconium was extremely significantly lower in term infants than in vernix caseosa (*p* < .001). Besides, there is no BCFA in meconium with carbon numbers between 12 and 16 (Figure 1b).

## 4 DISCUSSION

In our last study, we reported the content of BCFA in breast milk and its influencing factors, showing eight BCFA with a 0.55% cumulative concentration, and most BCFA are in the *sn*‐2 position. In this study, we studied and contrasted the FA composition of vernix caseosa and meconium in preterm infants and term infants. We further analyzed the impact of gestational age on BCFA in vernix caseosa and meconium compared to the Ran‐Ressler team, which found that BCFA were the main components of the intestinal contents of healthy newborns and had been a natural constituent present in the human gastrointestinal tract since a very early age and played an extraordinary role in the life cycle of humans. In addition, vernix caseosa and meconium are more common in the forms and material of BCFA than they are in breast milk. It was proposed that breast milk should only be used as the supplement to BCFA after birth, but may not completely meet the requirements of infant BCFA. Moreover, the content of BCFA both vernix caseosa and meconium was significantly higher in term infants than in preterm infants (*p* < .001).

The main BCFA in vernix caseosa and meconium are *iso*‐BCFA and *anteiso*‐BCFA, but there was a significant difference of the BCFA component between vernix caseosa and meconium. The distribution of common BCFA in vernix caseosa and meconium is shown in Figure 1. In contrast to vernix caseosa, the types and contents of most BCFA in meconium of both preterm and term infants were somewhat less than those in vernix caseosa. There was only one kind of *anteiso*‐BCFA (*anteiso*‐17:0) was detected in meconium. It was speculated that chain elongation is likely metabolic transformation; short‐chain BCFA may be transformed into long‐chain BCFA during gastrointestinal digestion. Therefore, it can also explain the concentration of *iso*‐22:0 in meconium exceeds its in vernix caseosa in the diagram. The *sn*‐2 BCFA content in vernix caseosa reached 14.42% ± 0.94%, including *iso‐*12:0, *iso‐*13:0, *anteiso*‐13:0, *iso*‐14:0, *anteiso*‐15:0, *iso*‐16:0, *anteiso*‐17:0, with a much smaller chain lengths variety. Among them, *iso*‐14:0, *anteiso*‐15:0, *iso*‐16:0 accounted for nearly 80% of BCFA in the primary BCFA. In vernix caseosa, the positional distribution of FA may affect their digestion and absorption. Once lipids join the small intestine, the action of pancreatic lipase hydrolysis forms FFA and *sn*‐2 MAG. They were well absorbed if BCFA were in the *sn*‐2 position, but when BCFA was in the *sn*‐1 and *sn*‐3 positions, they were hydrolyzed to FFA and produced insoluble saponified salts, which would severely decrease the rate of BCFA absorption, resulting in a loss of energy and calcium and increasing meconium hardness. This made BCFA's TAG synthesis at the *sn*‐2 position to eventually be used in infant formula milk powder could be the one based on in future research.

According to Friel et al. (1989), the meconium's wet weight in 27 term infants was about 8.9 g, and the average dry weight represents 32% of the wet weight, or 2.8 g. The results of this experiment indicated that about 8.08% are BCFA in term infants. Hence, the total content of BCFA was approximately 16.51 mg, which is very close to the result estimated by Ran‐Ressler (15.84 mg). The value, however, is much smaller than the amount of BCFA consumed by the fetus in the last month of pregnancy (fetal consumption of 6 mg BCFA per day, 180 mg BCFA in the last month of pregnancy) (Ran‐Ressler et al., 2008b, 2013), implying that a large amount of BCFA may be absorbed by the neonatal intestines.

There are two possible reasons for this; first, the lack of BCFA in the vernix caseosa of preterm infants leads to a decrease in the amount of BCFA consumed, resulting in low detection of BCFA in meconium. The second explanation may be that preterm infants have a higher medium and long‐chain BCFA absorption capacity than term infants, but are not prone to long‐chain BCFA, such as *iso‐*22:0, where the absorption capacity has decreased. It has been speculated that chain elongation is a likely metabolic transformation, and short‐chain BCFA may be transformed into long‐chain BCFA during gastrointestinal digestion (Mukherji et al., 2003; Terasaka et al., 1986).

The secretion of BCFA in amniotic fluid begins in the second trimester (>36 weeks), with a concentration of up to 17 mg/L (Egge et al., 1972). However, preterm infants (<37 weeks) rarely acquire BCFA at birth. Similarly, the reason for the low prevalence of breastfed infants is also associated with BCFA (Pisano et al., 2020; Ran‐Ressler et al., 2014; Underwood, 2013). Although there are few sources for adults to acquire BCFA in diets, they are a kind of trace lipids with high content in breast milk, with a range of up to 1.5 wt% (Hardman et al., 1999; Ran‐Ressler et al., 2013; Wang et al., 2020). At present, the composition of straight‐chain FA in infant formula milk powder is very similar to that of human breast milk. Some PUFA such as docosahexaenoic acid (DHA) and arachidonic acid (ARA) have also been added into infant formula, which have had a positive health effect on children.

Our previous studies showed there was a difference in the content of BCFA in lactating mothers' breast milk at different gestational ages and found that the BCFA in full‐term mothers' breast milk was higher than that of premature infants (Jie et al., 2018). The anti‐inflammatory effects of seven typical BCFA with different carbon chain length and branch chain position were studied (Yan et al., 2018). The results indicated that both *anteiso*‐BCFA and *iso*‐BCFA can inhibit the LPS presentation to the cells by inhibiting the expression of TLR‐4 on the surface of Caco‐2 cells, resulting in the decrease of IL‐8 and suppresses the IL‐8 mRNA expression in human intestinal epithelial cells. A recent animal test showed that BCFA are associated with the infants' gut microbiota and risk diseases, including necrotizing enterocolitis (NEC). The incidence of NEC in preterm infants may be multifactorial, and its pathogenesis is not completely clear to date. However, this is mostly associated with premature birth, feeding patterns, and intestinal flora colonization (Claud & Walker, 2001; Zhu et al., 2019). In most bacterial cell membranes, BCFA is a part. Exogenous BCFA intake can affect the colonization of intestinal flora and reduce the incidence of NEC in a neonatal rat pup model by 56% (Kaneda, 1991; Ran‐Ressler et al., 2011).

This research compared the BCFA of infants born at different gestational ages with vernix caseosa and meconium, showing an excess of BCFA in the vernix caseosa. The results indicated that BCFA are digested and absorbed in the infants' gastrointestinal tract. The content of BCFA in vernix caseosa and meconium of full‐term infants was significantly higher than that of preterm infants. BCFA may be considered a nutritional agent for the infants' intestinal health needs, warranting further study, especially in premature infants.

## CONFLICT OF INTEREST

The authors have no conflicts of interest to declare.

## AUTHOR CONTRIBUTION

**Weidi Li:** Data curation (equal); Methodology (lead); Writing‐original draft (lead). **Liang Jie:** Data curation (equal); Writing‐original draft (supporting). **Renqiang Yu:** Conceptualization (equal); Investigation (equal). **Qingzhe Jin:** Methodology (supporting). **Shanyu Jiang:** Investigation (equal). **Qitao Yin:** Conceptualization (equal); Funding acquisition (lead). **Wei Wei:** Conceptualization (equal); Project administration (equal); Writing‐review & editing (lead). **Xingguo Wang:** Project administration (equal); Supervision (lead).

## Supporting information

Supplementary Material

## Data Availability

The data that support the findings of this study are available from the corresponding author upon reasonable request.
