# Supplementary material for: Branched‐chain fatty acids in the vernix caseosa and meconium of infants born at different gestational ages
Source: Food Sci Nutr. 2021 May 6;9(7):3549–55. doi: 10.1002/fsn3.2306 (PMC8269679; doi:10.1002/fsn3.2306)
Supplement: Supplementary file 1 — Supplementary Material [file FSN3-9-3549-s001.docx]

*Supplementary Material*

Branched-chain Fatty Acids in the Vernix Caseosa and Meconium of Infants Born at Different Gestational Ages

Weidi Li ^a^, Liang Jie ^a^, Renqiang Yu ^b^, Qingzhe Jin ^a^, Shanyu jiang ^b^, Qitao Yin ^*b^, Wei Wei ^*a^ and Xingguo Wang ^a^

1. *Collaborative Innovation Center of Food Safety and Quality Control in Jiangsu Province, School of Food Science and Technology, Jiangnan University, Wuxi 214122, China.*
2. *The Affiliated Wuxi Maternity and Child Health Care Hospital of Nanjing Medical University, Wuxi 214002, China.*

* Corresponding author:

Qitao Yin, [Yqt1209@163.com](mailto:Yqt1209@163.com). Wei Wei, [weiw@jiangnan.edu.cn](mailto:weiw@jiangnan.edu.cn).


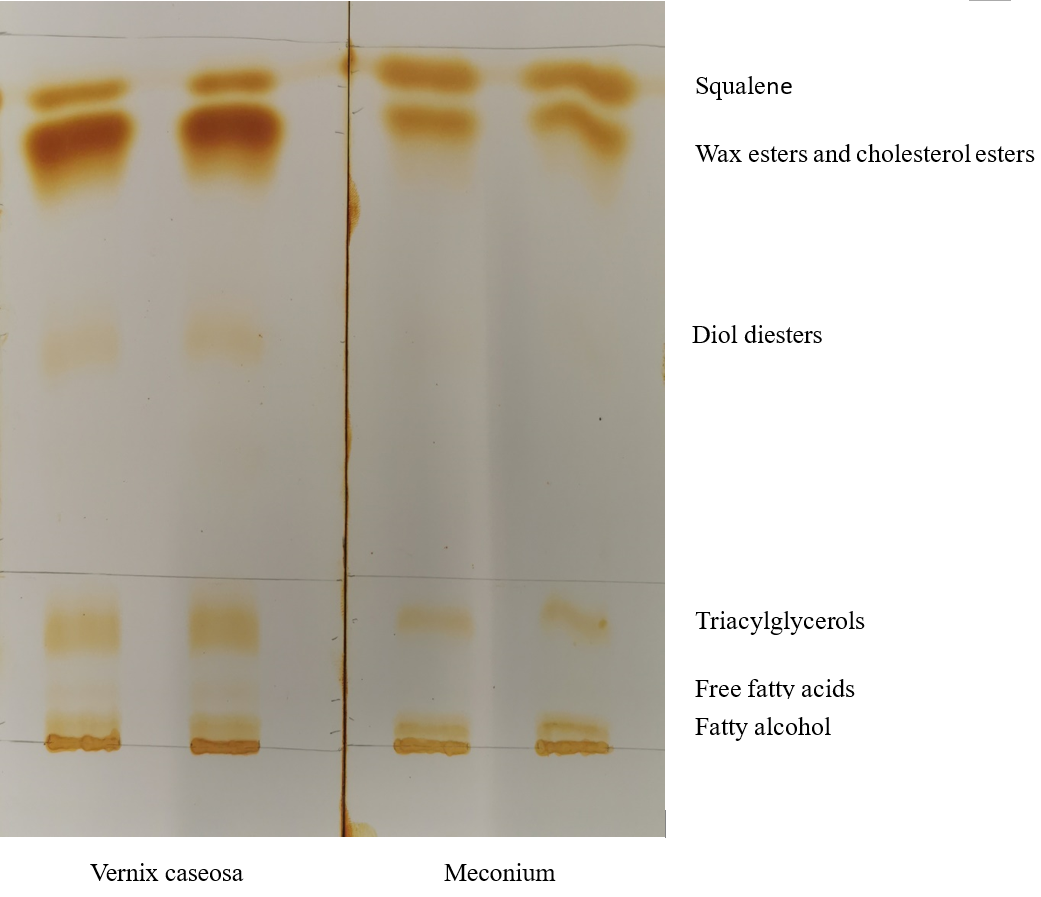


**Figure A1.** Lipid TLC separation of the vernix caseosa (a) and meconium (b).

Squalene (Rf 0.89-0.94), wax esters and cholesterol esters in one zone (Rf 0.66-0.74), diol diesters (Rf 0.46-0.52), triacylglycerols (Rf 0.19-0.27), free fatty acids (Rf 0.10-0.13), cholesterol esters (Rf 0.06-0.08), and polar lipids (Rf 0.00-0.01).

**Table A1**. Fatty acid composition in wax ester, triacylglycerols, *sn*-2 fatty acids of triacylglycerols, and free fatty acids of the vernix caseosa.

| Fatty acids | Wax ester | Triacylglycerols | | Free fatty acids |
| --- | --- | --- | --- | --- |
|  |  | Total | *sn*-2 position |  |
| 12:0 | 0.41 ± 0.03 | 0.43 ± 0.06 | 0.55 ± 0.11 | nd |
| 13:0 | nd | 0.62 ± 0.03 | 0.57 ± 0.13 | nd |
| 14:0 | 2.66 ± 0.22 | 15.29 ± 1.03 | 7.71 ± 0.20 | 6.67 ± 0.32 |
| 15:0 | 1.05 ± 0.12 | 7.99 ± 0.18 | 6.68 ± 0.39 | 4.48 ± 0.35 |
| 16:0 | 13.58 ± 2.34 | 27.18 ± 0.33 | 33.38 ± 0.98 | 32.08 ± 3.06 |
| 17:0 | 0.64 ± 0.29 | 0.44 ± 0.05 | 1.25 ± 0.21 | 1.14 ± 0.14 |
| 18:0 | 5.13 ± 0.58 | 1.57 ± 0.02 | 2.80 ± 0.30 | 11.28 ± 0.72 |
| 22:0 | 1.13 ± 0.55 | 0.03 ± 0.01 | nd | 1.28 ± 0.08 |
| 24:0 | 0.45 ± 0.14 | nd | nd | 1.72 ± 0.19 |
| 14:1 n-9 | 5.26 ± 0.51 | 2.33 ± 0.08 | 1.24 ± 0.11 | 0.81 ± 0.06 |
| 16:1 n-7 | 17.54 ± 0.86 | 1.40 ± 0.11 | 2.27 ± 0.16 | 2.77 ± 0.82 |
| 16:1 n-9 | 23.92 ± 2.09 | 18.63 ± 0.62 | 17.32 ± 0.39 | 11.85 ± 2.45 |
| 18:1 n-6 | 5.92 ± 1.01 | 7.68 ± 0.39 | 7.93 ± 0.45 | 6.17 ± 0.30 |
| 18:1 n-9 | 1.06 ± 0.09 | 3.32 ± 0.03 | 0.25 ± 0.04 | 0.70 ± 0.03 |
| 18:2 n-6 | 0.19 ± 0.62 | 0.34 ± 0.13 | 3.67 ± 0.35 | 1.18 ± 0.35 |
| *iso*-12:0 | nd | 2.62 ± 0.15 | 0.62 ± 0.12 | nd |
| *iso*-13:0 | nd | 0.54 ± 0.02 | 0.46 ± 0.02 | nd |
| *iso*-14:0 | 4.47 ± 0.15 | 5.05 ± 0.08 | 6.42 ± 0.46 | 3.49 ± 0.18 |
| *iso*-15:0 | nd | 0.30± 0.04 | nd | nd |
| *iso*-16:0 | 4.51 ± 0.05 | 3.45 ± 0.10 | 3.04 ± 0.17 | 3.61 ± 0.31 |
| *iso*-18:0 | 0.51 ± 0.04 | 0.25 ± 0.00 | nd | 0.70 ± 0.04 |
| *iso*-20:0 | 3.65 ± 0.42 | 0.37 ± 0.06 | nd | 0.80 ± 0.15 |
| *iso*-26:0 | nd | nd | nd | 1.41 ± 0.13 |
| *anteiso*-13:0 | nd | 0.37 ± 0.01 | 0.24 ± 0.04 | nd |
| *anteiso*-15:0 | 2.77 ± 0.08 | 1.94 ± 0.13 | 2.08 ± 0.22 | 1.32 ± 0.13 |
| *anteiso*-17:0 | 0.89 ± 0.04 | 0.70 ± 0.03 | 1.58 ± 0.20 | 0.49 ± 0.04 |
| 4-me-25:0 | 1.61 ± 0.15 | nd | nd | 0.93 ± 0.36 |
| 17-me-25:0 | 0.40 ± 0.07 | nd | nd | 2.31 ± 0.53 |
| ∑*iso*-BCFA | 13.14 ± 0.17 | 12.58 ± 0.07 | 10.53 ± 0.49 | 10.01 ± 0.47 |
| ∑*anteiso*-BCFA | 3.67 ± 0.11 | 3.01 ± 0.15 | 3.89 ± 0.45 | 1.81 ± 0.18 |
| ∑BCFA | 16.81 ± 0.12 | 15.59 ± 0.08 | 14.42 ± 0.94 | 11.82 ± 0.14 |

Data are represented as mean ± SD (n = 3); nd, not detected.
